# Decoding Representations of Scenes in the Medial Temporal Lobes

**DOI:** 10.1002/hipo.20960

**Published:** 2011-06-08

**Authors:** Heidi M Bonnici, Dharshan Kumaran, Martin J Chadwick, Nikolaus Weiskopf, Demis Hassabis, Eleanor A Maguire

**Affiliations:** 1 Wellcome Trust Centre for Neuroimaging, Institute of Neurology, University College London12 Queen Square, London, United Kingdom; 2 Institute of Cognitive Neuroscience, University College London17 Queen Square, London, United Kingdom; 3 Department of PsychologyJordan Hall, Building 01-420Stanford UniversityStanford, California; 4 Gatsby Computational Neuroscience Unit, University College London17 Queen Square, London, United Kingdom

**Keywords:** hippocampus, memory, MVPA, fMRI, pattern separation

## Abstract

Recent theoretical perspectives have suggested that the function of the human hippocampus, like its rodent counterpart, may be best characterized in terms of its information processing capacities. In this study, we use a combination of high-resolution functional magnetic resonance imaging, multivariate pattern analysis, and a simple decision making task, to test specific hypotheses concerning the role of the medial temporal lobe (MTL) in scene processing. We observed that while information that enabled two highly similar scenes to be distinguished was widely distributed throughout the MTL, more distinct scene representations were present in the hippocampus, consistent with its role in performing pattern separation. As well as viewing the two similar scenes, during scanning participants also viewed morphed scenes that spanned a continuum between the original two scenes. We found that patterns of hippocampal activity during morph trials, even when perceptual inputs were held entirely constant (i.e., in 50% morph trials), showed a robust relationship with participants' choices in the decision task. Our findings provide evidence for a specific computational role for the hippocampus in sustaining detailed representations of complex scenes, and shed new light on how the information processing capacities of the hippocampus may influence the decision making process. © 2011 Wiley Periodicals, Inc.

## INTRODUCTION

Ever since early studies of patient HM (Scoville and Milner,^1957^), it has been widely agreed that the hippocampus plays an important role in memory. Although its function has traditionally been viewed as limited to declarative memory (Squire et al.,^2004^), recent evidence suggests that it may also play a role outside this domain in tasks such as short-term memory (Ranganath and D'Eesposito,^2001^; Ranganath and Blumenfeld,^2005^), imagination (Hassabis and Maguire,^2007^, ^2009^), decision making (Eichenbaum,^2004^; Johnson et al.,^2007^: Kumaran et al.,^2009^) and even visual perception (Lee et al., 2005; Graham et al.,^2006^, ^2010^). Current perspectives, therefore, have emphasized that the function of the hippocampus, and indeed surrounding areas within the medial temporal lobe (MTL), may be best characterized by understanding the nature of the information processing they perform.

The application of multivariate pattern analysis (MVPA) techniques applied to functional magnetic resonance imaging (fMRI) data (Haynes and Rees,^2006^; Norman et al.,^2006^) offers the possibility of characterizing the types of neural representations and computations sustained by the human hippocampus, processes which are typically defined at the level of activity patterns across populations of neurons. Although conventional fMRI analyses typically focus on activity in each individual image voxel in isolation, MVPA utilizes information from patterns of activity expressed across multiple voxels, and hence large neuronal populations. Importantly, MVPA can infer the presence of neuronal representations previously thought to be below the spatial resolution of fMRI (Chadwick et al.,^2010^; Hassabis et al.,^2009^; Haynes and Rees,^2006^). Thus, the MVPA approach is useful not only because it reveals pattern information that is lost to conventional fMRI studies, but because it also permits the examination of patterns of fMRI activity associated with representations of *individual* stimuli.

In this study, we combined MVPA, high-resolution fMRI, and a simple decision making task to investigate scene processing within the MTL. Some theoretical accounts suggest that the hippocampus may play a relatively greater role in scene processing, as compared to other regions of the MTL which may largely subserve functions such as object processing (e.g., Bussey et al.,2002a, b; Cohen and Eichenbaum,^1993^; Eichenbaum,^2004^; Hassabis et al.,^2007^; Hassabis and Maguire,^2007^, ^2009^; Lee et al.,2005a, b). While neuropsychological evidence in patients with amnesia provides some support for this notion (Graham et al.,^2006^, ^2010^; Lee et al.,2005a, b; Bird et al.,^2008^; Hannula et al.,^2006^), existing fMRI evidence has tended to favor the hypothesis that the hippocampus plays a more domain-general role in memory (Diana et al.,^2008^; Preston et al.,^2010^).

**FIGURE 1:**
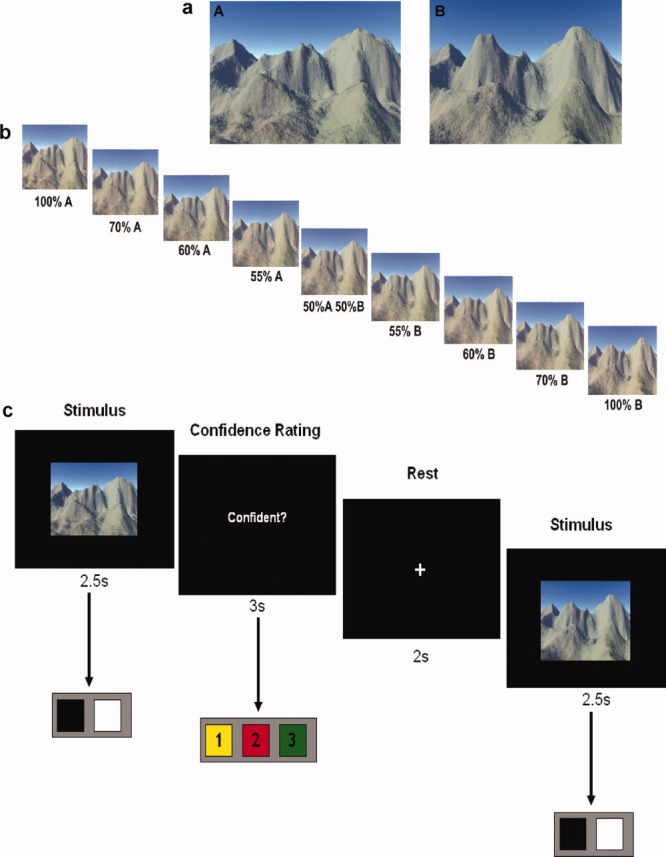
Stimuli and task. (a) The two original scenes - note they were not labeled A and B in the actual experiment. (b) The morph continuum proceeding from 100% scene A to 100% scene B. (c) A timeline of a single trial comprised a stimulus duration of 2.5 s during which the participant registered their decision. Participants then indicated their confidence in that decision during the next 3 s from a choice of not sure, fairly sure and very sure. There was a 2 s rest period before the next trial. [Color figure can be viewed in the online issue, which is available at wileyonlinelibrary.com]

In this study, prior to scanning, participants learnt which action was rewarded (e.g., right button press) in relation to a given scene (e.g., A), receiving monetary feedback for appropriate responses. Importantly, the two scenes that were employed (A, B: although they were not labeled as such during the experiment; see Fig. 1a) were highly similar, sharing multiple features, allowing us to examine the role of the hippocampus in maintaining distinct representations of individual scenes through pattern separation. During scanning, participants viewed the original scenes (100% A and 100% B), as well as morphed scenes spanning a continuum from A to B (e.g., 30% A, 40% A—see Fig. 1b). Although the morphs themselves were not linked to reinforcement at any stage, participants were instructed to select the action most likely to yield monetary reward, while no feedback was provided.

We set out to address three specific issues of interest: first, do patterns of activity in the MTL contain information that allows the identity of individual scenes to be decoded? Second, are scene representations in the hippocampus more distinct than that elsewhere in the medial temporal lobe (MTL), consistent with the operation of pattern separation computations in the hippocampus? Third, when current sensory input is ambiguous, most acutely in 50% morph trials, do patterns of activity in the hippocampus systematically relate to participants' choices? If so, this would demonstrate that hippocampal activity is not purely stimulus-driven, rather it is likely to reflect an interaction between sensory inputs and mnemonic representations, a process which may bias participants' choice and therefore contribute to the decision making process.

## METHODS

### Participants

Sixteen healthy right-handed participants (8 male) took part in the experiment (mean age 24.4 years, standard deviation (SD) 2.8, range 21–30). All had normal or corrected-to-normal vision and gave informed written consent to participation in accordance with the local research ethics committee.

### Stimuli

The two scenes (A and B, see Fig. 1a) were created using Terragen, version 0.9.43 for windows (http://www.planetside.co.uk). Scene A was created first, and then modified to create scene B. Several phases of piloting ensured that the two scenes were regarded as highly similar while being distinct and were approximately equated for the number of constitute elements and overall complexity. Once the two scenes were created, seven morphed scenes were generated using Morph Age, version 4 for Mac (http://www.creaceed.com/morphage). Seven morphs were generated to proceed in a continuous fashion from scene A to B (70% A and 30% B, 60% A and 40% B, 55% A and 45% B, 50% A and 50% B, 45% A and 55% B, 40% A and 60% B, 30% A and 70% B). As the morph levels approached 50%, more features from the two original stimuli become shared, increasing the ambiguity (see Fig. 1b).

### Prescan Training

Participants were aware that they would receive a monetary reward for their correct answers, while wrong answers lost money. Prior to scanning, participants learnt which action was rewarded (e.g., action A—right button press) in relation to a given stimulus (i.e., scene A), receiving monetary feedback for appropriate actions. The two scenes that were used (A, B) were never labeled as such during the experiment. During this phase, participants were presented with scene A or B one at a time each for 2.5 s. Allocation of button press was switched for half of the participants. In each trial they were given feedback informing them if their choice was correct or incorrect. To ensure that choice performance had stabilized before scanning, each participant performed at least 20 trials during this phase, although all reached criterion (10 correct responses in a row) well before this (see Results). Next, the morph stimuli as well as the original scene stimuli were presented in pseudorandom order, each scene shown for 2.5 s, and three times during the course of the session. Once again participants were instructed to choose the action most likely to yield reward given the composition of the scene being viewed; no feedback was given. Following each trial they were asked to provide a confidence rating about the choice they had just made: 1 = not sure, 2 = fairly sure, and 3 = very sure. After this learning phase, participants repeated phase one, viewing the original two scenes again to ensure behavioral performance was stabilized before scanning.

### During Scanning

During scanning, participants saw the two scenes, 100% A and 100% B, as well as the seven morphed stimuli one at a time in a pseudorandom order ensuring there were no biases towards either scene A or B (see example trial timeline in Fig. 1c). Stimuli were presented 40 times each. As before, participants were instructed to choose the action most likely to yield reward given the composition of the scene being viewed, and then to provide a confidence judgement. No feedback was given during the scanning phase of the experiment, although participants were instructed that they would be paid in proportion to their performance on the task, at the end of the experiment.

### Postscan Debriefing

After scanning, each participant was debriefed. They were first asked to perform a probe test, where 40 stimuli were presented in the same format as the scanning task. Stimuli consisted of 20 scenes based on 100% scene A and 20 based on 100% scene B. In each case the stimulus was exactly the same as the original scene, but with successive shifts in view angle of 5 degrees, either to the right or the left. Altogether there were 10 scene A stimuli shifted to the right, 10 shifted to the left, and 10 scene B stimuli shifted to the right, 10 shifted to the left. The aim of this task was to explore the nature of the strategies used during the discrimination task. If participants were able to select the correct action in response to rotated versions of the original scenes this would suggest that behavioral performance was based on view-independent scene representations, rather than the sampling of individual features. Finally, each participant was asked to draw what he/she could remember of the two scenes (100% A and 100% B).

### Scanning Parameters

#### Functional MRI

Data were acquired in a partial volume focused on the temporal lobes. A 3-T Magnetom Allegra head only MRI scanner (Siemens Medical Solutions, Erlangen, Germany) operated with the standard transmit-receive head coil was used to acquire the functional data with a T2*-weighted single-shot echo-planar imaging (EPI) sequence (in-plane resolution = 1.5 × 1.5 mm^2^; matrix = 128 × 128; field of view = 192 × 192 mm^2^; 35 slices acquired in interleaved order; slice thickness = 1.5 mm with no gap between slices; echo time TE = 30 ms; asymmetric echo shifted forward by 26 phase-encoding (PE) lines; echo spacing = 560 μs; repetition time TR = 3.5 s; flip angle α = 90°). All data were acquired at 0° angle in the anterior-posterior axis. An isotropic voxel size of 1.5 mm × 1.5 mm × 1.5 mm was chosen for an optimal trade-off between BOLD sensitivity and spatial resolution. Further, the isotropic voxel dimension reduced resampling artifacts when applying motion correction. To ensure optimal data quality, images were reconstructed online and underwent online quality assurance (Weiskopf et al.,^2007^). For distortion correction (Hutton et al.,^2002^), field maps were acquired with a standard manufacturer's double echo gradient echo field map sequence (TE = 10.0 and 12.46 ms, TR = 1,020 ms; matrix size = 64 × 64), using 64 slices covering the whole head (voxel size 3 mm × 3 mm × 3 mm).

#### Structural MRI

A whole brain 3D FLASH sequence was acquired with a resolution of 1 mm × 1 mm × 1 mm. In addition, high-resolution structural images were acquired on a 3-T whole body MRI scanner (Magnetom TIM Trio, Siemens Medical Solutions, Erlangen, Germany) operated with the standard transmit body coil and 32-channel head receive coil. Images were acquired in a partial volume focused on the temporal lobes. A single-slab 3D T2-weighted turbo spin echo sequence with variable flip angles (SPACE; Mugler et al.,^2000^) in combination with parallel imaging was used to simultaneously achieve a high-image resolution of ∼500 μm, high sampling efficiency and short scan time while maintaining a sufficient signal-to-noise (S/N) ratio (SNR). After excitation of a single axial slab the image was read out with the following parameters: resolution = 0.52 × 0.52 × 0.5 mm^3^, matrix = 384 × 328, partitions = 104, partition thickness = 0.5 mm, partition oversampling = 15.4%, field of view = 200 × 171 mm^2^, TE = 353 ms, TR = 3,200 ms, GRAPPA × 2 in phase-encoding (PE) direction, bandwidth = 434 Hz/pixel, echo spacing = 4.98 ms, turbo factor in PE direction = 177, echo train duration = 881, averages = 1.9. For reduction of signal bias due to, e.g., spatial variation in coil sensitivity profiles, the images were normalized using a prescan and a weak intensity filter was applied as implemented by the scanner's manufacturer. To improve the SNR of the anatomical image, four scans were acquired for each participant, coregistered and averaged.

Manual segmentation of the hippocampus (HC), entorhinal cortex (EC), and parahippocampal gyrus (PHG) was performed with the ROI module of the Anatomist software (http://brainvisa.info/index.html) on the T2 high-resolution structural images. EC and PHG were segmented according to the protocol described in Insausti et al. (1998). The anatomy of the hippocampus was identified using Duvernoy (2005). These segmentations generated a set of masks for each participant for each hemisphere. Within each of these masks the number of 1.5 mm^3^ voxels from the EPI images were (averaged across the two hemispheres): HC 1093.47 (96.37), EC 252.19 (79.82), and PHG 277.13 (122.92).

### fMRI Analyses

#### Univariate analysis

A standard mass univariate statistical analysis was performed using SPM5 (http://www.fil.ion.ucl.ac.uk/spm). The first six EPI volumes were discarded to allow for T1 equilibration effects (Frackowiak,^2004^). Spatial preprocessing consisted of realignment and normalization to a standard EPI template in Montreal Neurological Institute (MNI) space, and smoothing using a Gaussian kernel with FWHM of 8 mm. After preprocessing, statistical analysis was performed using the general linear model. Each trial was modeled by a delta function defined using the event onset, and this was convolved with the canonical hemodynamic response function to create a regressor for each stimulus type. Therefore, there were nine regressors (the two original scenes 100% A and 100% B, and the seven morphed scenes). Participant-specific movement parameters were included as regressors of no interest. Participant-specific parameter estimates pertaining to each regressor (betas) were calculated for each voxel. These parameter estimates were entered into a second level random-effects analysis using a one-way analysis of variance (ANOVA), with the nine scene regressors as the factors. Given our a priori interest in the medial temporal lobes, a significance threshold of *P* < 0.001, uncorrected for multiple comparisons, was used for voxels within this region. A significance threshold of *P* < 0.05 corrected for family-wise errors was used for voxels elsewhere in the partial volume. We conducted this univariate analysis as an initial step prior to proceeding to a multivariate approach.

We examined all possible comparisons between trial types (72 in all, e.g., 100%A >100%B, 100%A >60%A, and so on). We factored in and out reaction times, confidence ratings, correct and incorrect trials, at the standard uncorrected threshold of *P* < 0.001, and the even more liberal *P* < 0.05 uncorrected. We found no significant effects. This was also the case when a parametric analysis was employed across the range of morphs. These null univariate results were expected because the conventional univariate approach works by measuring gross voxel activity differences between conditions. With all conditions (i.e., stimuli) involving identical processes, it is not surprising that this method did not reveal any significant differences, hence the advantage of using a multivariate approach.

#### Image preprocessing for multivariate analysis

Multivariate preprocessing was performed using SPM5. The first six EPI volumes were discarded to allow for T1 equilibration effects (Frackowiak,^2004^). The remaining EPI images were realigned to correct for motion effects, and coregistered to the whole brain 3D FLASH structural scan, after which the EPI volumes and whole brain FLASH structural scan were coregistered to the high resolution T2 structural scans. Each EPI volume was minimally smoothed with a 3-mm FWHM Gaussian kernel. Each trial was then modeled as a separate regressor, where the time of display of each trial was modeled as an event and convolved with the canonical hemodynamic response function. Participant-specific movement parameters were included as regressors of no interest. Participant-specific parameter estimates pertaining to each regressor (betas) were calculated for each voxel. The voxel size used by the classifier was 1.5 × 1.5 × 1.5 mm^3^.

#### Multivariate classification

We used a two-step procedure incorporating first feature selection and then final multivariate classification (Guyon and Elisseeff,^2003^). The purpose of feature selection is to reduce the set of features (in this case, voxels) in a dataset to those most likely to carry relevant information. This is effectively the same as removing voxels most likely to carry noise and is a way of increasing the S/N ratio. Feature selection can therefore greatly improve the performance of multivariate pattern classification (Guyon and Elisseeff,^2003^).

The particular feature selection strategy employed was a multivariate searchlight strategy, which assesses the local pattern of information surrounding each voxel in turn (Kriegeskorte et al.,^2006^; Hassabis et al.,^2009^; Chadwick et al.,^2010^; see feature selection section below for more details). The overall classification procedure involved splitting the imaging data into two segments: a “training” set used to train a linear support vector machine (SVM; Duda et al.,^2001^) (with fixed regularization hyperparameter *C* = 1) to identify response patterns related to the stimuli being discriminated, and a “test” set used to independently test the classification performance.

In the example, here we will focus on discrimination between the two original scenes (100% A and 100% B). Similar procedures were followed in each of the analyses described in the main text. Prior to multivariate classification, feature selection was performed on the data from the *training* set. This step produced a subset of voxels within the hippocampus (or in EC/PHG) that contained the greatest amount of scene information within the training dataset. Using this voxel subset, the SVM classifier was, for example, trained to discriminate between the two scenes using the “training” image dataset and tested on the independent “test” dataset. The classification was performed with a SVM using the LIBSVM (http://www.csie.ntu.edu.tw/∼cjlin/libsvm/) implementation. We used a standard k-fold cross-validation testing regime (Duda et al.,^2001^) wherein *k* equaled the number of experimental trials, with the data from each trial set aside in turn as the test data, and the remaining data used as the training set (on each fold, the feature selection step was performed using only data from this training set). This therefore generated k sets of SVM training and test sets that produced overall classification accuracy from the proportion of correct classification “guesses” across all *k* folds of the cross-validation.

#### Feature selection

Feature selection was implemented using a multivariate searchlight strategy (Kriegeskorte et al.,^2006^), which examines the information in the local spatial patterns surrounding each voxel within the search space. Thus, for each voxel within the chosen anatomical region of interest, we investigated whether its local environment contained information that would allow accurate decoding of two scenes. For a given voxel, we first defined a small sphere with a radius of three voxels centered on the given voxel. This radius was chosen because previous demonstrations of decoding using hippocampal fMRI activity and this searchlight method used radius three (Hassabis et al.,^2009^; Chadwick et al.,^2010^). Note that the “spheres” were restricted so that only voxels falling within the given region of interest were included. Therefore, the shape of the “sphere,” and the number of voxels within it varied depending on the proximity to the region of interest's borders.

A linear SVM was then used in order to assess how much scene information was encoded in these local pattern vectors. This was achieved by splitting the feature selection data set into a training set and a test set (again it is important to note that all of the data used in this feature selection step is derived from the *training* set of the overall classification procedure, and therefore is fully independent of the final classification). The training set was then used to train a SVM classifier using the LIBSVM (http://www.csie.ntu.edu.tw/∼cjlin/libsvm/) implementation and a fixed regularization hyper parameter of *C* = 1. We used a standard k-fold cross-validation testing regime (Duda et al.,^2001^) wherein *k* equaled the number of experimental trials minus one (as one trial is already removed for use as the overall testing set—see above), with the data from each trial set aside in turn as the test data, and the remaining data used as the training set. This therefore generated *k* sets of SVM training and test sets that produced overall classification accuracy from the proportion of correct classification “guesses” across all *k* folds of the cross-validation. This procedure was repeated for each searchlight sphere, thus generating a percentage accuracy value for every single voxel within the search space.

The searchlight analysis described above therefore produces an “accuracy map” of the given ROI, with an accuracy value at each voxel representing the amount of decoding information contained within the searchlight sphere surrounding that voxel. This allows us to perform feature selection by selecting searchlight spheres with high accuracy values. In this case, the searchlight with the maximal accuracy value was chosen as the output of feature selection. In cases where more than one searchlight carried the maximal accuracy value, all voxels from all the maximal searchlight spheres were included as the feature selection output.

## RESULTS

### Behavioral Results

Prior to scanning, participants learnt to select the appropriate action for each scene (A, B), taking an average of 5.5 trials (SD 5.97) to reach criterion (10 correct responses in a row). To ensure that choice performance had stabilized before scanning, each participant performed at least 20 trials during this phase. Participants then received practice on the scene morphs task, in order to familiarize them with each of the seven morph scenes, and to ensure that behavioral performance had stabilized before scanning (see Methods).

**FIGURE 2:**
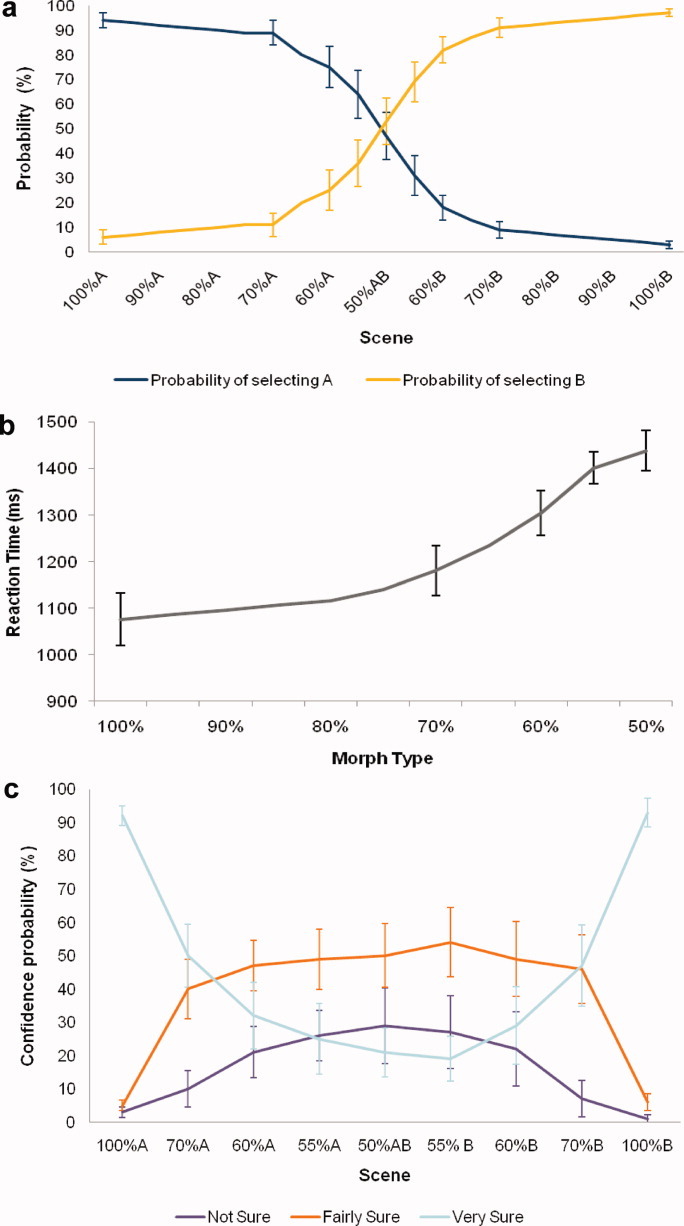
Behavioral data. (a) The psychometric functions for accuracy for the 16 participants showed a sigmoid profile. (b) Participants were less accurate and slower with increasing noise in the sensory input. (c) Participants' pattern of confidence ratings also followed the expected distribution. Morphs approaching the two original scenes were afforded higher confidence ratings, and more ambiguous morphs lower ratings. [Color figure can be viewed in the online issue, which is available at wileyonlinelibrary.com]

During scanning, participants viewed both original scenes (100% A, B), as well as the seven morph scenes a total of 40 times each, randomly intermixed. While participants were not provided with feedback during scanning, they were instructed to choose the action most likely to yield reward given the composition of the scene being viewed, and rate their level of confidence in their choice (see Fig. 1c). The psychometric function for accuracy for the 16 participants showed a sigmoid profile (Fig. 2a). Further, participants were slower and less accurate with increasing noise in the sensory input (Fig. 2b), consistent with previous suggestions that decisions under perceptual uncertainty reflect the accumulation of evidence towards a threshold (Gold and Shadlen,^2007^). Participants' pattern of confidence ratings also followed the expected distribution. Morphs approaching the two original scenes were afforded higher confidence ratings, and more ambiguous morphs lower ratings (Fig. 2c). Of note, even when the perceptual input was entirely ambiguous (i.e., 50% morphs), participants tended to rate their decisions with a moderate degree of confidence, on average, rather than a subjective sense of guessing. Behavioral accuracy (*P* = 0.40), reaction times (*P* = 0.19), and confidence ratings (*P* = 0.35) did not change significantly over the course of scanning.

Following the scanning session, participants took part in a postexperimental testing session that provided ancillary information concerning the nature of the strategies used during the discrimination task (see Methods). This revealed that in general participants were able to select the correct action in response to rotated versions of the original scenes suggesting that behavioral performance was based on view-independent scene representations, rather than the sampling of individual features (mean: 26.5; SD 4.56). All but two participants performed significantly above chance on this task. When these two participants were removed from the analyses described below, there was no change to any of the findings. In addition, all participants were able to draw the main features of scenes A and B, and could note the differences between the two.

### Neuroimaging Results

#### Scene specific information is present in the hippocampus and MTL under conditions of perceptual certainty (i.e., 100% A, 100% B)

**FIGURE 3:**
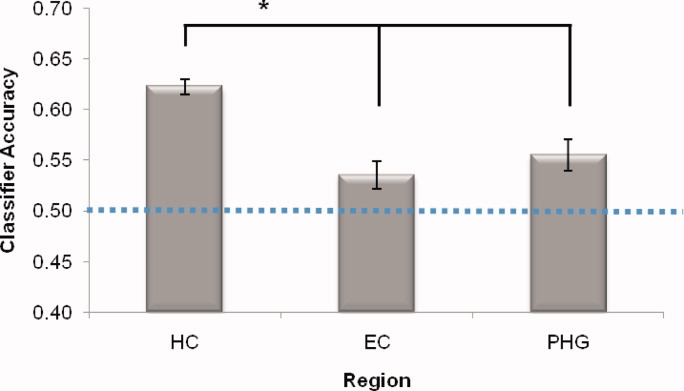
Average classifier accuracy values for 100% scenes. Hippocampus (HC), entorhinal cortex (EC) and parahippocampal gyrus (PHG) results for discriminating between the 100% scene trials. All were significantly above chance, with the HC classifier performing significantly better than EC and PHG classifiers. [Color figure can be viewed in the online issue, which is available at wileyonlinelibrary.com]

We first asked whether patterns of activity in the MTL distinguished been the two original scenes, providing evidence for the coding of scene-specific information in this region. As expected, a standard univariate fMRI analysis failed to yield significant results (see Methods). Hence, we carried out an MVPA analysis in which a classifier for each region of interest was trained on part of the 100% A and 100% B scene trials, labeled according to participants' choices. The classifiers' performance was then tested on an unseen portion of trials (see Methods). Each classifier produced an accuracy value for each region of interest in each hemisphere in every participant. We performed one-way repeated-measure Analysis of Variance (ANOVA) with hemisphere as a factor to test for any interactions between the hemispheres. None were found in any analysis and therefore the results we report are collapsed across hemisphere. Each MTL region was able to distinguish the two scenes with an accuracy value significantly above chance (all *P*< 0.05; see Fig. 3). These results provide evidence that neural representations that contain information about the scene currently being experienced are present in the hippocampus, and surrounding MTL, under conditions of perceptual certainty.

Given that participants were performing a decision task, a key question, however, is whether these patterns of activity comprise neural representations of the currently viewed scene (i.e., scene A), or instead retrieved motor actions (e.g., right button press). To address this issue, we again trained classifiers based on each of the relevant regions of interest on 100% A, B trials, labeled according to participants' decisions. For this analysis, however, the classifier was tested on trials where morph stimuli (e.g., 50%) were presented, also labeled according to participants' responses. If retrieved motor actions drive the ability of the classifier to decode participants' decisions in relation to 100% scenes, one would predict that a classifier trained on 100% morphs would perform similarly when tested on 50% trials. In fact, classifier accuracies in hippocampus and elsewhere in this additional analysis were not significantly different from chance (all *P* > 0.1), rendering it unlikely that motor variables contribute significantly to the decoding of currently viewed scene. It is also worth noting that the rewarding outcome associated with both scenes was identical, excluding the possibility that neural representations of the outcome itself could contribute to classification accuracy.

#### Scene representations are more patterns separated in the hippocampus, than upstream in MTL

We next looked for differences between MTL regions. This analysis revealed that classification accuracy under perceptual certainty (i.e., 100% A, B) was significantly higher in the hippocampus (*F*_2, 126_ = 12.33, *P* < 0.001; Fig. 3), compared to the other two regions of interest within the MTL (HCvEC: *P* < 0.001; HCvPHG: *P* < 0.001). These results provide evidence that scene representations in the hippocampus are more distinct, or orthogonal, than those just one synapse upstream in EC. As such, the observed findings provide evidence that the hippocampus performs pattern separation, acting to orthogonalize similar input patterns that arrive fromthe EC, resulting in neural representations coding for complex scenes that are more distinct than those in neighboring brain regions.

#### Patterns of activity in the hippocampus correlate with participants' choices under perceptual ambiguity during morph trials

Having obtained evidence that the hippocampus supports distinct scene representations where perceptual input is complete, we next turned our attention to the neuroimaging data during trials where morph scenes were viewed. In this way, we set out to ask whether patterns of activity in the hippocampus during viewing of morph scenes correlate with participants' subsequent choices. We focused primarily on 50% morph trials since perceptual input under these circumstances is entirely ambiguous.

**FIGURE 4:**
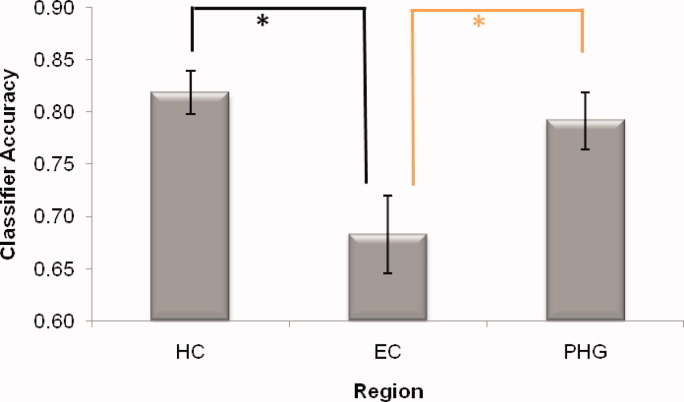
Average classifier accuracy values for 50% morphed scenes. HC, EC, and PHG results for classifying participants' decisions. All were significantly above chance, with HC and PHG classifiers both performing significantly better than EC. [Color figure can be viewed in the online issue, which is available at wileyonlinelibrary.com]

A classifier for each region of interest was trained on part of the 50% morph scene trials, which were labeled according to participants' choices. The classifier's performance was then tested on an unseen portion of 50% morph trials (see Methods). As in the previous analysis, each classifier produced an accuracy value for each region of interest in each hemisphere for every participant. The classifiers' ability to assign correct labels to test trials was significantly above chance in each MTL region (all *P* < 0.001; see Fig. 4).

We also looked for differences between MTL regions. An interaction was found between regions (*F*_2, 24_ = 9.242, *P* = 0.001; Fig. 4) driven by the hippocampus and parahippocampal gyrus performing significantly better than the EC (HCvEC: *P* < 0.001; PHGvEC: *P* < 0.01).

This result, in addition to subjective reports of relatively high confidence, demonstrates that participants' choices, even on 50% trials, were associated with systematic patterns of activity within the MTL. Given that hippocampal activity patterns under these conditions cannot be purely stimulus-driven, we asked whether they might instead reflect categorical decisions (e.g., that the stimulus looks “A-like”) or retrieved motor actions (e.g., right button press). We addressed this issue in an analysis where a classifier was trained on 50% morphs, labeled according to participants' responses, and tested on 100% scene trials. This analysis found chance levels of classification in the hippocampus and other MTL regions (all regions *P* > 0.1), demonstrating that the activity patterns in these regions are unlikely to reflect the coding of decision or action variables. Instead, a parsimonious account of why hippocampal activity patterns observed in 50% morph trials correlate with participants' choices is that they arise from an interaction between external sensory inputs (i.e., 50% morph stimulus) and internal stored representations (e.g., corresponding to the 100% A and B scenes), which systematically influences the decision-making process.

Given that hippocampal activity patterns during 50% morph trials cannot merely be a consequence of the current stimulus input, and instead would seem to arise through an interaction between external sensory inputs and internal stored representations, it is interesting to ask what specific computations might be responsible. One possible scenario is that hippocampal activity patterns during a given 50% morph trial reflect reinstatements of the entire original learnt representation (e.g., 100% scene A), a process consistent with the operation of a bistable attractor network, and one that might plausibly be viewed to induce participants to choose one response over another. Indeed neural network models have often favored the idea that memories are stored as discrete local attractors (Hopfield,^1982^; Rolls and Treves,^1994^), with partial or ambiguous inputs (50% morphs) abruptly inducing the network to occupy one state (e.g., relating to 100% A) or the other (e.g., relating to 100% B) through “global” pattern completion (i.e., output of entire stored pattern). However, the failure of a classifier trained on 50% morphs to correctly assign labels to 100% scenes (see previous analysis), argues strongly against this scenario. Furthermore, the complementary analysis where the classifier was trained on 100% scenes, and tested on 50% morphs (see above), provides further support for this conclusion.

While attractor networks provide an elegant computational solution to how memories are stored and retrieved in the face of noisy (i.e., uncertain) external inputs, our failure to find evidence of discrete attractors within the hippocampus is not entirely surprising given previous work in rodents where the operation of attractors may depend on the exact experimental parameters imposed (Leutgeb et al.,^2005^; Wills et al.,^2005^). We therefore considered an alternative scenario, where hippocampal ensembles respond to partial inputs (i.e., 50%) by transitioning to intermediate network configurations (e.g., relating to a 70% morph) via a more limited form of pattern completion. To address this issue, we asked whether a classifier trained on 50% morph trials would generalize successfully (i.e., correctly assign labels to test trials) to other morphs trials (i.e., 55–70%). Interestingly, this was the case in the hippocampus, but not in the parahippocampal gyrus (*F*_2, 22_ = 6.729, *P* < 0.01; HCvEC: *P* < 0.01; HCvPHG: *P* < 0.01; PHGvEC: *P* = 0.59). This result suggests, therefore, that partial inputs (i.e., morph trials) lead to the expression of neural representations in the hippocampus which share similarity to one another, but differ from those expressed under perceptual certainty (100% A, B).

## DISCUSSION

This study used MVPA together with high-resolution fMRI to investigate scene processing within the MTL. Our findings demonstrate that scene-specific information was represented across the MTL, and not just solely in the hippocampus. Importantly, however, we observed that scene-specific patterns of activity in the hippocampus were more distinct than in upstream MTL cortices. Furthermore, we demonstrate that under conditions of perceptual uncertainty, a classifier trained on patterns of activity in the hippocampus, could decode participants' ultimate choices. Our results, therefore, provide evidence that the hippocampus sustains distinct representations of complex scenes through pattern separation, thereby dovetailing with previously observed scene discrimination deficits in rodents and humans with hippocampal damage (Graham et al.,^2006^; McHugh et al.,^2007^). Moreover, this study, in highlighting a robust link between patterns of neural activity in the hippocampus and choice behavior, provides new insights into how its specific representational and computational capacities may be brought to bear on the decision making process.

Our results show that under conditions of perceptual certainty (i.e., 100% A, B) patterns of activity throughout the MTL, including the hippocampus, contain information concerning the scene currently being viewed, which permitted successful decoding by a classifier. Importantly, we were able to link the decoding ability of the classifier to the composition of the perceptual input itself (i.e., 100% A vs. B), since classification performance based on participants' responses (e.g., right or left response) was not significantly different from chance levels. This suggests, therefore, that MTL representations code the currently viewed scene itself, rather than information relating to the chosen action. While other studies have examined category level specificity (scene vs. object) in different MTL regions (Diana et al.,^2008^; Preston et al.,^2010^), our study goes beyond prior work in providing evidence that neural representations that differentiate between individual scene exemplars (as opposed to categories) are present in the hippocampus and across the MTL.

Critically, our data demonstrate that patterns of activity coding highly similar scenes are more distinct in hippocampus than elsewhere in MTL. This result implies that hippocampal codes are more orthogonal than those immediately upstream (in EC), providing strong support for a hippocampal role in pattern separation. Computational models have emphasized the ability of the hippocampus to minimize interference between similar memories, mediated by the properties of the dentate gyrus (DG) which support the formation of sparse conjunctive codes based on inputs from EC (McNaughton,^1987^; O'Reilly and McClelland,^1994^; McClelland et al.,^1995^). While recording data in rodents has provided evidence that neural representations in DG are indeed more orthogonal than those upstream (Leutgeb et al.,^2007^), evidence supporting a similar role for the human hippocampus has been relatively lacking. This is because previous work investigating the nature of information processing carried out by the human hippocampus has typically used conventional univariate fMRI analysis methods where BOLD signal at the level of individual voxels is compared across different experimental conditions (Kumaran and Maguire,^2006^; Bakker et al.,^2008^). Since computations such as pattern separation (and completion) are typically defined at the level of patterns of activity across populations of neurons, it has been difficult to draw definitive conclusions from the results of such univariate fMRI studies (see Kumaran and Maguire,^2009^). For instance, a recent study concluded that DG/CA3 performs pattern separation because this subregion showed a relatively greater BOLD response to lure objects (i.e., distorted versions of previously seen objects), as compared to CA1 (Bakker et al.,^2008^). As has been argued elsewhere, while this finding may indeed reflect pattern separation in DG/CA3, the univariate nature of the analysis procedure employed does not permit plausible alternative accounts to be discounted (e.g., a role for this region in mismatch detection - see Kumaran and Maguire,^2009^).

Taken together, our results suggest that while the MTL may sustain relatively coarse codes of individual scenes, the hippocampus supports more complex and distinct scene-specific representations, implemented through its ability to rapidly form sparse conjunctive codes from incoming input patterns (i.e., from upstream MTL cortices; Cohen and Eichenbaum,^1993^). As such, our findings accord with previous work suggesting that hippocampal damage in both humans (Graham et al.,^2006^), and rodents (McHugh et al.,^2007^) produces a specific deficit in the ability to behaviorally discriminate between highly similar scenes, or environments. In one study, mutant mice, which had been genetically engineered to show a specific deficit in DG dependent pattern separation, were able to correctly appreciate the significance of two very different environments (McHugh et al.,^2007^). Critically, however, discrimination ability was significantly impaired when the contexts were made highly similar, a behavioral deficit that was associated with a failure of neural pattern separation in the hippocampus.

Our experimental paradigm, which can be considered a synthesis of previous work in the fields of hippocampal research (Leutgeb et al.,^2005^; Wills et al.,^2005^; Graham et al.,^2006^) and perceptual decision making (Shadlen and Newsome,^2001^; Romo and Salinas,^2003^; Heekeren et al.,^2008^), also enabled us to demonstrate the intimate relationship between patterns of activity in the hippocampus and choice behavior, when external perceptual inputs were held constant (i.e., 50% morph trials). These results therefore imply that patterns of neural activity observed in the hippocampus during uncertainty are not a faithful reflection of the absolute sensory properties of the environment, as is viewed to occur in low level visual regions like MT (Shadlen and Newsome,^2001^; Gold and Shadlen,^2007^). Importantly, we also demonstrate that classifier decoding under such circumstances does not reflect the explicit coding of decision (e.g., categorizing a given stimulus as “A-like”) or motor (e.g., button pressed) variables, processes often viewed in the context of perceptual decision making tasks to be instantiated in cortical regions such as DLPFC and motor cortices, respectively (Heekeren et al.,^2008^). Our results, therefore, suggest that patterns of activity in the hippocampus under these conditions may arise through an interaction of external sensory inputs and internal stored representations, a process that may plausibly involve a limited form of pattern completion. Our finding that partial inputs (i.e., morph trials) lead to the expression of neural representations in the hippocampus which share similarity to one another, but differ from those expressed under perceptual certainty (100% A, B) argue against discrete attractors in the hippocampus. Instead these data are broadly consistent with the notion that hippocampal ensembles may occupy intermediate network configurations spanning the morph continuum, transitioning between them through limited pattern completion.

Extensive theoretical research has considered how the unique information processing capacities of the hippocampus might relate to its pivotal role in memory across species. Our study provides neural evidence, based on the synthesis of MVPA techniques and high-resolution fMRI technology, in support of specific representational and computational functions of the human hippocampus, and provides insights into how these processes might play out in a simple decision making scenario. However, the precise computations by which these hippocampal activity patterns are generated, and the exact nature of their contribution to the decision making process, remain open questions. In the future, it will be important to further explore how hippocampal processing relates to ultimate behavioral choice, and determine whether this involves interactions between the hippocampus and other brain regions such as the prefrontal cortex and striatum.
